# Single Nucleotide Polymorphisms Can Create Alternative Polyadenylation Signals and Affect Gene Expression through Loss of MicroRNA-Regulation

**DOI:** 10.1371/journal.pcbi.1002621

**Published:** 2012-08-16

**Authors:** Laurent F. Thomas, Pål Sætrom

**Affiliations:** 1 Department of Cancer Research and Molecular Medicine, Norwegian University of Science and Technology, Trondheim, Norway; 2 Interagon AS, Laboratoriesenteret, Trondheim, Norway; 3 Department of Computer and Information Science, Norwegian University of Science and Technology, Trondheim, Norway; Center for Genomic Regulation, Spain

## Abstract

Alternative polyadenylation (APA) can for example occur when a protein-coding gene has several polyadenylation (polyA) signals in its last exon, resulting in messenger RNAs (mRNAs) with different 3′ untranslated region (UTR) lengths. Different 3′UTR lengths can give different microRNA (miRNA) regulation such that shortened transcripts have increased expression. The APA process is part of human cells' natural regulatory processes, but APA also seems to play an important role in many human diseases. Although altered APA in disease can have many causes, we reasoned that mutations in DNA elements that are important for the polyA process, such as the polyA signal and the downstream GU-rich region, can be one important mechanism. To test this hypothesis, we identified single nucleotide polymorphisms (SNPs) that can create or disrupt APA signals (APA-SNPs). By using a data-integrative approach, we show that APA-SNPs can affect 3′UTR length, miRNA regulation, and mRNA expression—both between homozygote individuals and within heterozygote individuals. Furthermore, we show that a significant fraction of the alleles that cause APA are strongly and positively linked with alleles found by genome-wide studies to be associated with disease. Our results confirm that APA-SNPs can give altered gene regulation and that APA alleles that give shortened transcripts and increased gene expression can be important hereditary causes for disease.

## Author Summary

Variants in DNA that affect gene expression—so-called regulatory variants—are thought to play important roles in common complex diseases, such as cancer. In contrast to variants in protein-coding regions, regulatory variants do not affect protein sequence and function. Instead, regulatory variants affect the amount of protein produced. The 3′ untranslated region (UTR) is one gene region that is critically important for gene regulation; cancers for example, often express genes with shortened 3′UTRs that, compared with full-length 3′UTRs, have higher and more stable expression levels. We have investigated one kind of regulatory variant that can affect the 3′UTR length and thereby cause disease. We identified several such variants in different genes and found that these variants affected the genes' expression. Some of these variants were also strongly linked with known markers for disease, suggesting that these regulatory variants are important hereditary causes for disease.

## Introduction

In protein-coding genes, the polyadenylation process consists of cleaving the end of the 3′ untranslated region (UTR) of precursor messenger RNA (pre-mRNA) and adding a polyadenylation (polyA) tail. Alternative polyadenylation (APA) can occur when several polyadenylation (polyA) signals lie in the last exon of a protein-coding gene. Many APA signals are evolutionary conserved [1], and Expressed Sequence Tag (EST) data suggest that 54% of human genes have alternative polyadenylation signals [1]. The polyA signals themselves are hexamer DNA sequences that usually lie 10 to 30 nucleotides upstream from the cleavage site [2], but a GU-rich region 20 to 40 nucleotides downstream of the cleavage site is also important for the polyA-process [2].

One functional consequence of APA is transcripts with different 3′UTR lengths and different microRNA (miRNA) regulation [3], [4]. Shortened transcripts tend to have increased expression compared with longer transcripts, and the same expression increase can be achieved by deleting miRNA target sites in non-shortened transcripts [5].

Data on APA can be used as an efficient biomarker for distinguishing between cancer subtypes and for prognosis [6], and seems to play an important role in gene deregulation and in many human diseases [7]. One such mechanism for deregulation is mutations in the polyA signal or GU-rich downstream region [7]. A single nucleotide polymorphism (SNP) in the GU-rich region downstream of an alternative polyA signal in the *FGG* gene has for example been shown to affect the usage of this polyA site, and has been associated with increased risk for deep-venous thrombosis [8]. Similarly, a mutation in the 3′UTR of the *CCND1* gene has been shown to create an alternative polyA signal and is associated with increased oncogenic risk in mantle cell lymphoma [9].

**Figure 1:**
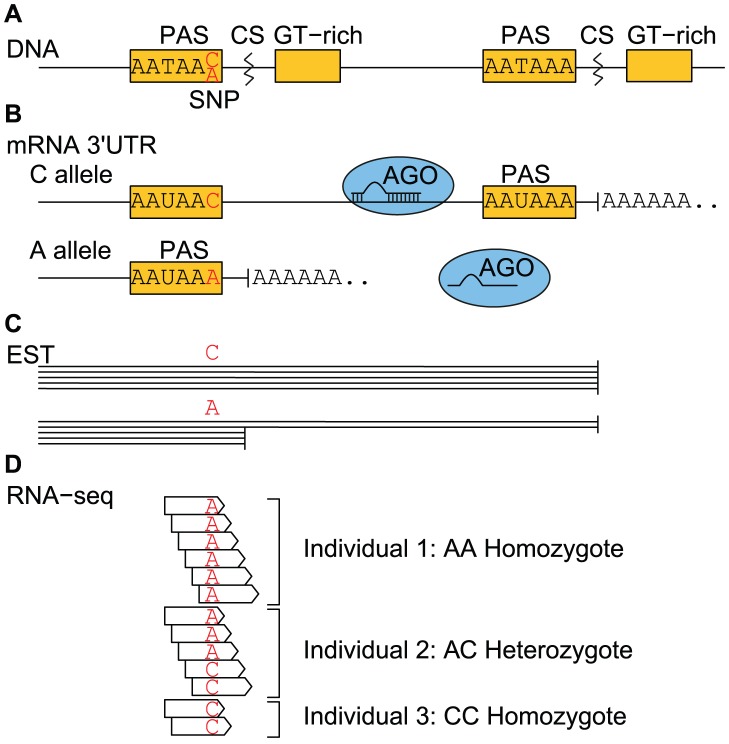
A model of the effect of APA-SNPs in the 3′UTR of a gene. (A) For the C allele, the second cleavage site (CS) is used, because the first polyA signal (PAS) is not functional. For the A allele, the first PAS is functional, therefore the pre-mRNA can be cleaved at the first CS, resulting in a loss of functional miRNA target sites downstream (indicated with loss of Argonaute (AGO) binding), and increased gene expression (B). (C) EST sequences enable identifying APA-SNP alleles and 3′UTR length. (D) RNA-seq reads enable genotyping APA-SNPs and quantifying expression patterns.

Hypothesizing that mutations in DNA elements such as the polyA signal can be an important cause of altered APA, we investigated to what extent SNPs can create or disrupt APA signals (APA-SNPs). Specifically, we tested whether APA-SNPs can give shorter 3′UTRs, increased gene expression through loss of miRNA regulation (Fig. 1), and be associated with disease. Our hypothesis focuses on shorter 3′UTRs rather than longer ones, because the loss of functional miRNA sites in the 3′UTR is more likely than the gain of new sites downstream of the gene.

First, by analysing EST data, we found that SNPs can create polyA motifs and affect 3′UTR length. Second, differential allelic expression from RNA-seq data, as well as mRNA and miRNA microarray expression data revealed an association between alternative polyA site strength (signal and GU-content), loss of miRNA target sites, and transcript expression. Third, based on these analyses we also identified significant APA-SNPs. Fourth, we mapped the identified SNPs to disease-associated SNPs and found that APA alleles were significantly correlated with disease-risk alleles. Together, these results suggest that APA-SNPs can be a significant causative mechanism in disease (Fig. S1).

## Results

### SNPs can create and delete polyadenylation signal motifs

The distribution of SNPs within 3′UTRs is fairly uniform [10] (Fig. S2A). The main exceptions are microRNA target sites and the start and end of the 3′UTR, which have decreased SNP diversity that is consistent with these regions containing functional elements under selective pressure [10]. Indeed, when specifically investigating the region around the transcription end site, we found that the position containing the polyA signal has a markedly decreased SNP density (Fig. S2B,C), indicating that SNPs arising there could have a high functional impact.

To analyse SNPs in alternative polyadenylation signals, we first identified a set of SNPs that potentially create new APA signals in 3′UTRs. Specifically, we searched for any Hapmap SNP [11] that could create or disrupt one of the 13 known polyA signal hexamers [1] in any coding gene's 3′UTRs (see Methods). We found 1954 SNPs, including 755 SNPs that are mono-allelic in the CEU population from Hapmap [11] (see Datasets). We kept only the APA-SNPs that change from no signal to one signal in the locus, by discarding loci with several signals in the 40 nucleotides around the SNP, discarding SNPs that change one signal into another, and discarding mono-allelic SNPs. After filtering, 412 SNPs that can create or delete potential polyadenylation signals remained. We will from now refer to them as our candidate SNPs.

### EST data indicate that SNPs can give functional alternative polyA sites

To investigate whether SNPs can create functional alternative polyA sites, we analysed the EST-based polyA sites from the PolyA_Db database [12], [13]. In the PolyA_Db database, there are several polyA sites which do not have any noticeable polyA signal (according to the reference genome) in the 40, 80, and 100 nucleotides upstream from the reported cleavage site position (Table S1). In those regions, we used different SNP data to look for SNPs that could create a polyA signal with the non-reference allele. When considering regions of 100 nucleotides and SNPs from NCBI dbSNP Build 130 [14], we could identify polyA signals with the alternative allele for more than 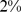 of the missing signals. Some of the remaining sites can probably be explained by SNPs further upstream, and some other by exon splicing, by alterations in ESTs that are not registered in dbSNP, or as false positive sites.

**Figure 2:**
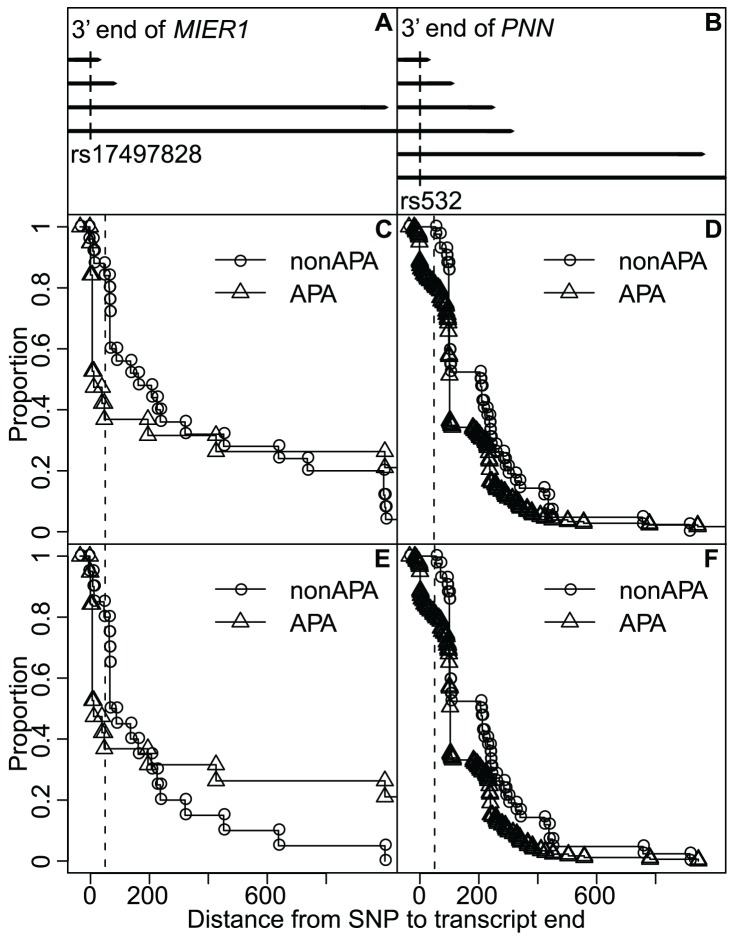
SNPs can affect 3′UTR length. Panels (A) and (B) show 3′ ends of the *MIER1* and *PNN* genes as annotated in PolyA_Db (3′ ends of the horizontal lines), and their candidate APA SNP. The four other graphs show the inverse cumulative distribution of EST sequence ending position for APA alleles (triangles) and non-APA alleles (circles). The dashed vertical line shows the threshold separating short and long transcripts. The transcript proportion is decreasing before the threshold for APA alleles, compared to non-APA alleles. This decrease indicates that APA alleles are more likely to produce shorter transcripts. Panels (A), (C) and (E) show the *MIER1* gene. Panels (B), (D) and (F) show the *PNN* gene. Several unknown alleles could be imputed through haplotypes (included in Panels (C) and (D)).

Since EST-based annotated polyA sites can be affected by SNPs, we wanted to test whether alleles in polyA sites could be associated with EST ending positions. Specifically, we first took the intersection between the polyA signals from our 412 candidate SNPs, and the polyA sites from PolyA_Db database [12], [13]. We identified 18 intersecting polyA sites that have a polyA signal for either the reference or the non-reference allele. These sites corresponded to 18 SNPs in 18 genes. Five SNPs were discarded because they lie within the 20 last nucleotides of the reference 3′UTR. The following 13 genes remained: *ABCC4, AKAP13, FANCD2, KY, MIER1, OSTM1, PNN, RASGRP3, RHOJ, SELS, SHMT1, SLBP*, and *SLC11A2*. Second, for each of these genes, we identified and imputed (see Methods) alleles at the SNPs in the EST sequences when possible, and tested if the proportion of alleles with polyA signal (APA alleles) was different for EST sequences ending within the interval 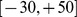 nucleotides around the polyA site, compared to EST sequences ending further downstream (see Methods). The two genes *MIER1* (SNP rs17497828) and *PNN* (SNP rs532) were significant (Fig. 2, Table 1). After correcting for multiple testing (Benjamini & Hochberg correction), the genes remained significant when including alleles imputed based on haplotype (Table 1).

**Table 1:** Significant genes in the EST analysis.

	no correction		Benjamini&Hochberg correction	
Gene	imputation	no imputation	imputation	no imputation
*MIER1*	0.004*	0.016*	0.032*	0.103
*PNN*	0.005*	0.004*	0.032*	0.058

* shows significant p-values.

P-values for 2×2 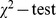 comparing the proportion of alleles with APA signal for short versus long EST sequences. The *MIER1* and *PNN* genes were significant (including and not including imputed alleles). After correcting for multiple testing, the proportions including imputed alleles remained significantly different between short and long ESTs.

For *MIER1*, 12 of the 16 EST sequences ending near the annotated APA site had the APA allele (including 2 with a clear polyA tail), whereas 3 had the non-APA allele (none of them had a clear polyA tail). Similarly, for *PNN*, all of the 34 EST sequences ending near the annotated APA site had the APA allele (including 10 with a clear polyA tail). Together, these results suggest that SNPs can create functional APA sites and thereby affect 3′UTR length.

### RNA-seq data indicate that SNPs in polyA sites can affect transcript length and give increased transcript expression

EST data can be used to identify alleles and transcript ending positions (Fig. 1), but EST data seldom have sufficient resolution to quantify transcript expression levels. In contrast, RNA-seq data can both be used to genotype SNPs [15] and to analyse transcript length and expression patterns. The main challenge with RNA-seq data compared with ESTs, however, is the shorter sequence reads, which makes it challenging to distinguish between homozygotes, heterozygotes with strong expression differences between its alleles (allelic imbalance), sequencing errors, and alignment errors.

To explore whether RNA-seq data could reveal whether APA-SNPs affect transcript expression, we therefore developed and validated an RNA-seq-based genotyping approach (see Supporting Text S2). We then used this approach to show that APA-SNPs can affect transcript expression and that this effect is associated with loss of miRNA regulation. Specifically, we first show that homozygous APA-SNPs have significantly shorter 3′UTRs than have heterozygous or homozygous wildtype SNPs. Second, we show an association between allelic imbalance of heterozygous APA-SNPs and the two following important features of polyA sites: signal strength and GU level downstream of the cleavage site. Third, we show that the loss of miRNA target sites can be the missing link in this association. Fourth, we use allelic imbalance to detect potentially functional APA-SNPs. Fifth, we show that APA-SNPs at strong sites (strong APA signal and high GU level) that have a strong predicted effect on miRNA regulation, have higher allelic imbalance and higher transcript expression than have other APA-SNPs.

#### Transcripts are shorter for genes with homozygous APA-SNPs

RNA-seq data give the opportunity to both genotype exonic SNPs and determine transcript structure. We therefore decided to use the Burge RNA-seq data to determine whether APA-SNPs had a significant effect on transcript length. Moreover, as the Burge RNA-seq data set consists of samples from both highly proliferating cell lines and highly differentiated human tissues and as transcripts in proliferating cells tend to have shorter 3′UTRs, we also wanted to determine the effect that the cell's proliferation status had on transcript length. Specifically, we first estimated for each gene and RNA-seq sample, the transcript's 3′ end position and its distance from the 3′ end of the longest annotated transcript (see Methods). Second, we divided the RNA-seq samples into two groups such that the five cancer cell lines and one immortalized cell line were defined as proliferating, whereas the 16 other tissue samples were defined as non-proliferating.

Third, from the 412 candidate APA-SNPs, we discarded those that share the same gene and those that lie upstream of the longest 3′UTR (to avoid combinations of alternative splicing and alternative polyadenylation), resulting in 362 SNPs. In total, 262 unique SNPs had 3′ end estimates and genotypes available (6852 data points). To analyse the impact of APA-SNPs on 3′ end positions, we only considered SNPs that lie far enough (at least 1500 bp upstream) from the annotated 3′end. This final requirement gave 93 unique SNPs (2340 data points).

Fourth, we ran correlation analyses between the genotype (WT homozygous: 0, heterozygous: 1, and APA homozygous: 2) and the negative logarithm of the distance between the estimated and the annotated transcript end (see Methods). As expected, we found a significant negative correlation (Pearson's correlation coefficient 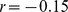, p-value 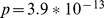, sample size 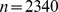), which shows that APA homozygotes are shorter than the WT ones. Then, we tested the correlation between the negative log distance and the proliferation status of the cell types (proliferating: 1; non-proliferating: 0). Again, as expected, we found a significant negative correlation (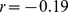, 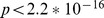, 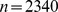). When sub-grouping the samples based on proliferation status (Fig. S3), we could not detect a significant genotype correlation in the proliferating cells—possibly because transcripts are already short in these cells due to other factors. For non-proliferating cells, however, we found that APA homozygotes were significantly shorter than the two other genotypes (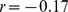, 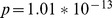, 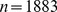). This result confirms our previous EST results that APA SNPs can affect transcript length.

#### Heterozygous SNPs affecting strong polyA sites have an increased imbalance towards APA alleles

Since RNA-seq data can genotype our candidate SNPs and at the same time determine transcript expression levels, we decided to analyse ratios of allele expression (allelic imbalance). According to our hypothesis (Fig. 1), APA alleles can shorten transcripts, resulting in loss of miRNA targeting and increased transcript expressions. To test this hypothesis, we investigated allelic imbalance of our APA-SNPs in 19 of the samples from the Burge RNA-seq data; we excluded the three samples (MAQC, MAQC UHR, and MD435) that were a mixture of several individuals. We expected increased transcript expression for the APA allele compared to the non-APA allele; that is, a positive log ratio of the APA allele expression over the non-APA allele expression. Moreover, we expected this allelic imbalance to depend on two important polyA site features: polyA signal strength and downstream GU level.

**Figure 3:**
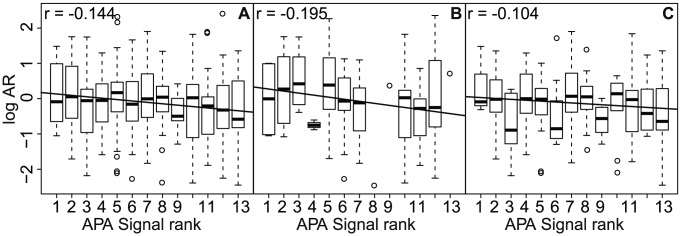
Increased allelic imbalance correlates with signal strength and depends on downstream GU-content. Log allelic ratio distribution of APA allele over non-APA allele for each polyA signal ordered by strength. Panel (A): log allelic ratio is negatively correlated with signal rank for all APA-SNPs. Compared with all APA-SNPs, APA-SNPs with a GU-rich region (Panel (B)) have a stronger negative correlation between log allelic ratio and signal rank. For APA-SNPs without a GU-rich region (Panel (C)), there is no significant correlation between signal rank and log allelic ratio. The graphs include data from the 19 non-mixed cell lines and tissues. The line in each panel shows the linear regression line; the corresponding Pearson correlation coefficient 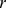 is in the panel's upper left corner.

Some polyadenylation signals occur more frequently upstream of known polyA sites than other signals do [1]. By assuming that this frequency of occurrence correlate with signal strength, such that frequent signals have a higher probability of causing polyadenylation than have rare signals (Table S4), we expected that frequent (strong) signals would have a higher allelic ratio (AR) than rare (weak) signals. We compared the distribution of allelic ratios of APA allele over non-APA allele for each signal, ordered by strength rank such that strong (frequent) signals had a low rank. As expected, we found that signal rank is negatively correlated with log allelic ratio (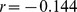, 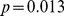, 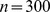) (Fig. 3A). Strong signals tend to have high and positive log AR; that is, a higher expression of the APA allele than of the non-APA allele. This fits our hypothesis that transcripts with an APA allele can escape miRNA targeting, resulting in increased gene expression.

In addition to having strong polyA signals, functional polyA sites tend to have a GU-rich region downstream of the cleavage site [2]. We therefore expected that SNPs creating alternative polyadenylation signals with a GU-rich region downstream of the signal had a higher allelic imbalance than the ones with no particular GU-rich region.

We computed the GU level for each of our candidate SNPs. As the background value outside the GU-rich region is about 0.51 (Fig. S4), we used a threshold of 0.55 to define SNPs that have a downstream GU-rich region. Then, in each of the two GU groups, we investigated the allelic ratio distribution for each signal. We still found a negative correlation between the signal rank and the log allelic ratio for the SNPs with a GU-rich region (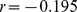, 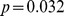, 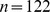) (Fig. 3B). In contrast, for the SNPs without a GU-rich region, log AR did not correlate with signal rank (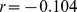, 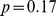, 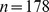) (Fig. 3C). This indicates that increased allelic imbalance at APA-SNPs requires both a strong signal and a GU-rich downstream region.

**Figure 4:**
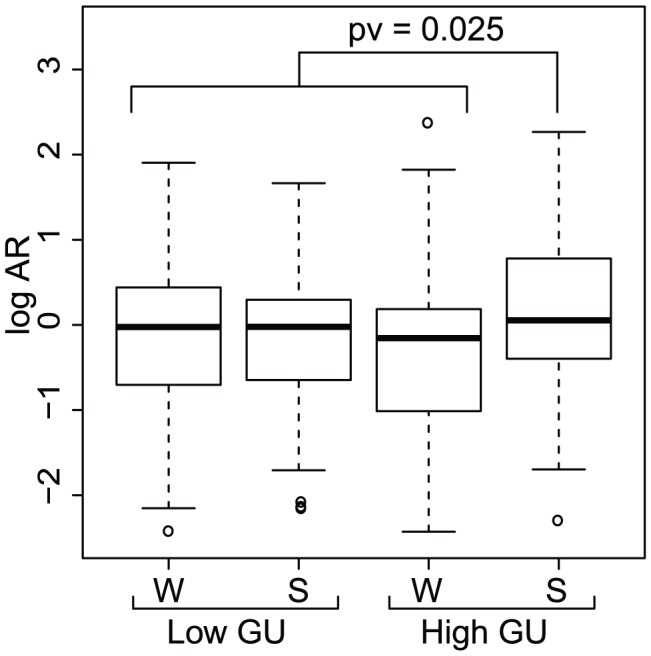
Allelic imbalance distributions according to signal strength and downstream GU levels. Allelic imbalance is increased towards APA alleles for APA-SNPs in strong (S) signals with high downstream GU levels. The graph shows a box-plot of the log AR distribution of APA-SNPs grouped by signal strength (weak (W) and strong (S)) and downstream GU levels.

To further evaluate the connection between signal strength, GU level, and allelic imbalance, we grouped the SNPs according to their GU level and their signal strength (Fig. 4; strong: 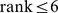; weak: 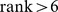). Compared with the other three groups, APA-SNPs with a strong signal and a GU-rich region had a significantly higher mean and median log AR (Student's t-test, 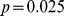; Wilcoxon rank sum test, 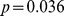). Together, these results suggested that alternative polyadenylation can give increased expression of APA alleles.

#### The loss of miRNA target sites can explain an important part of allelic imbalance

Increased expression of APA allele transcripts is consistent with loss of miRNA regulation, but other factors such as RNA-binding proteins could potentially also explain these results. We therefore wanted to test whether loss of miRNA regulation could be a significant factor in the increased allelic imbalance. Specifically, we matched the miRNA expression data of the MCF7, BT474, and T47D breast cancer cell lines from Landgraf *et al.* [16] with the allelic ratios from the corresponding cell lines from the Burge dataset (24 unique SNPs, 34 allelic imbalance values in the 3 cell lines). Given the miRNA profile of the considered cell line, we then for each SNP computed a score which predicted the potential effect that a cleavage site at the SNP locus would have on miRNA regulation (see Methods). Finally, we ran several linear regression analyses with the log allelic ratios as response variable and the signal rank, the GU level, and the miRNA score difference as dependent variables.

Basic linear models with signal rank, GU level, or miRNA score alone showed that these variables could explain 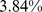, 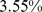, and 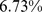 of the response variance, respectively. A model with signal rank and GU level decreased the partial explained variance for each of the two variables compared to the two individual models. In contrast, adding the miRNA variable to the Signal rank model, or the GU level model increased all partial 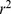 values, indicating that the dependent variable is a conjunction of these variables. Similarly, adding the miRNA variable to the Signal rank+GU level model could increase all the partial 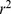 as well. In that full model, the miRNA variable could explain 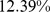 of the response variance (p-value 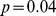). This indicates that loss of miRNA target sites can partly explain the increased allelic imbalance for APA-SNPs in strong APA signals with high downstream GU content.

#### Allelic expression can detect potentially functional APA-SNPs

Having established that APA-SNPs can give allelic imbalance by affecting miRNA regulation, we set out to identify functional APA-SNPs. We identified SNPs from the 19 non-mixed samples from the Burge dataset that were classified as heterozygous when mapping reads to both the reference and non-reference allele-based genomes and that had at least 10 allele counts in total. This resulted in 75 individual/SNP pairs (36 unique SNPs), which we tested individually for significant positive imbalance; that is, an APA allele count significantly greater than the non-APA allele count. We used a 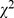 goodness-of-fit test (1 degree of freedom) to test if the allele counts fit the hypothesis of an equal proportion. Three heterozygotes were significant and after correcting for multiple testing by using the Benjamini & Hochberg correction, two heterozygotes remained significant. The two individual/SNP pairs had both a positive log-ratio, a GU-rich region and a strong APA signal. After correcting with the more stringent Bonferonni method, the same two pairs remained. Those two individual/SNP pairs were actually the same SNP (rs2269123 in gene *MRPS34*) from two breast cancer cell lines (BT474 and MCF-7; p-values 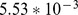 and 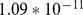, respectively), suggesting that this SNP gives a functional APA signal that strongly affects host gene expression.

#### MicroRNAs link higher proportion of APA alleles to higher gene expression

Since heterozygous SNPs in strong APA signals can have an increased imbalance towards APA alleles, we investigated whether positive allelic imbalance can be associated with increased gene expression; that is whether a higher proportion of APA alleles than non-APA alleles was associated with an increased total allele count. We focused on the 12 samples from the Burge dataset that we could match to miRNA expression data in similar cell types from Landgraf *et al.* [16]; these were the 3 breast cancer cell lines (MCF7, BT474 and T47D), and the liver, heart, testis, and 6 cerebellum samples. In those 12 samples, we identified 174 allelic ratios (97 unique SNPs) that were classified as heterozygous when mapping to both the reference and non-reference allele based genomes. Given the miRNA profile, we then assigned a miRNA score which predicted the potential effect that a cleavage site at the SNP locus would have on miRNA regulation (see Methods).

**Figure 5:**
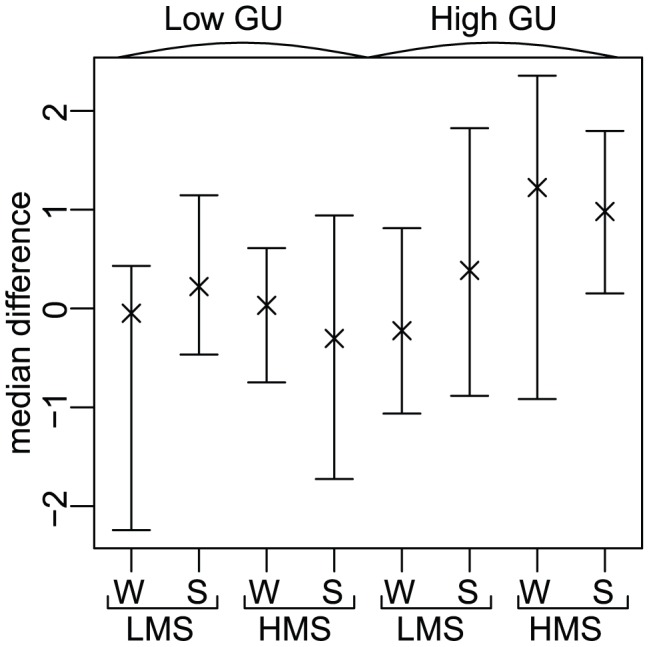
SNP expression difference between SNPs with positive and negative log allelic ratios. Logarithm of SNP expression median difference between SNPs with positive log allelic ratios and those with negative log allelic ratios, in several groups (low and high GU level, low (LMS) and high (HMS) miRNA score, and weak (W) and strong (S) signal). Crosses show median differences. Bootstrapping median differences gives 95% CI. Only one CI does not contain zero: the one with high GU, HMS and S, indicating that positive allelic imbalance for SNPs in strong polyA sites and affecting miRNA target sites, is associated with increased SNP expression, and therefore increased gene expression.

**Figure 6:**
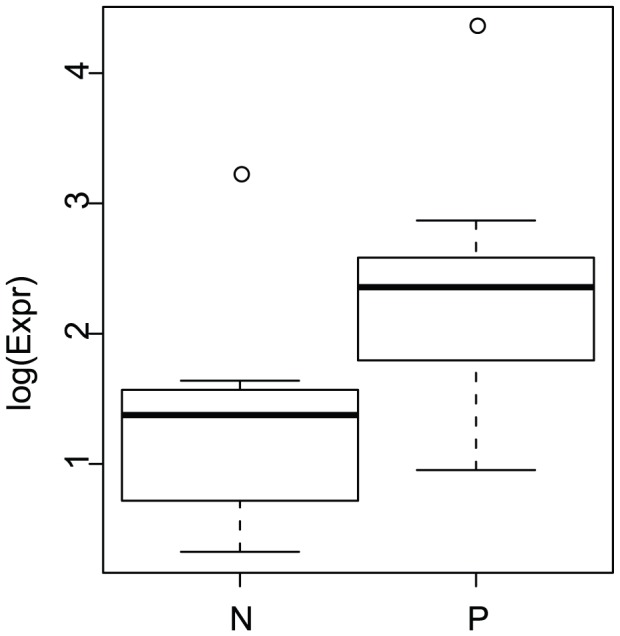
SNP expression distributions according to allelic imbalance direction. SNPs in strong APA signal, with high GU level and high miRNA score, have a significantly higher logarithm of SNP expression for SNPs with imbalance towards APA allele (positive (P) log allelic ratio), compared to SNPs with imbalance towards non-APA allele (negative (N) log allelic ratio).

Based on the 174 allelic ratios, we compared SNP expression (sum of APA and non-APA allele counts) for groups with higher APA allele proportion (positive log AR) with groups with higher non-APA allele proportion (negative log AR; Fig. 5). We found that SNPs with strong APA signal, high GU level, and high miRNA score had a significant log SNP expression difference between positive log ratios and negative log ratios. This indicates that APA alleles of SNPs with strong APA sites and high miRNA scores can upregulate gene expression (Fig. 6). This links positive allelic imbalance to higher gene expression.

### MicroRNAs link genotype to increased gene expression

To confirm the results from the RNA-seq-based allelic imbalance analyses, we turned to gene expression data from the well characterised Hapmap population. We looked at human gene expression profiling of EBV-transformed lymphoblastoid cell lines from 270 unrelated Hapmap individuals [17], and genotypes of the same individuals, from the Hapmap database [11]. Specifically, we first investigated whether genotypes of SNPs in strong polyA sites that affect miRNA targeting in general are associated with increased gene expression. Second, we investigated whether individual APA-SNP genotypes correlate significantly with gene expression.

#### Genotype of SNPs in strong polyA sites and the loss of miRNA target sites can explain increased gene expression

From the Hapmap expression profiles and our 412 potential APA-SNPs, we identified 333 SNPs that could be mapped to 315 unique probe IDs. Discarding SNPs sharing the same probe IDs, resulted in 299 unique SNPs and probe IDs. We then used human miRNA expression profiles from EBV-transformed lymphoblastoid cell lines [18], to compute a miRNA score that quantifies the potential effect of a polyA cleavage site at each SNP locus on miRNA regulation (see Methods).

Simple regression analyses with mRNA expression as response variable and with each of genotype, signal rank, local GU level downstream of the signal, and miRNA score as dependent variables, found that the GU level explained the most of the mRNA variance (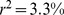). We therefore computed the GU level in the whole 3′UTR and ran a regression of the mRNA expression on this variable. Surprisingly, we found that this variable was positively correlated with higher gene expression for our 299 genes (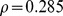, 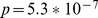) and could explain 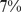 of the response variance. One possible explanation is that non-canonical polyA sites are thought to rely mostly on downstream GU-rich elements [19]. If this explanation is true we could expect that genes with increased GU level in 3′UTR can have a higher number of APA sites, which could result in generally higher mRNA expression. Indeed, based on polyA_Db, we found that 3′UTR GU level is positively correlated with the number of polyA sites in each gene (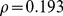, 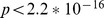, 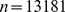). Moreover, the number of polyA sites is also positively correlated with mRNA expression from microarray data (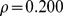, 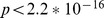, 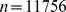). Expectedly, longer 3′UTRs are more likely to have more polyA sites (correlation coefficient 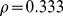, 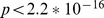, 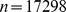). However, we also found that the GU level is correlated with 3′UTR length (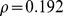, 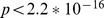, 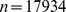). All these results suggest that the 3′UTR GU level is a confounding variable giving increased APA and thereby mRNA expression. We therefore analysed mRNA expression data after correcting for the general 3′UTR GU level; *i.e.* we regressed the mRNA expression on the 3′UTR GU content and used the residuals as the new response variable.

**Figure 7:**
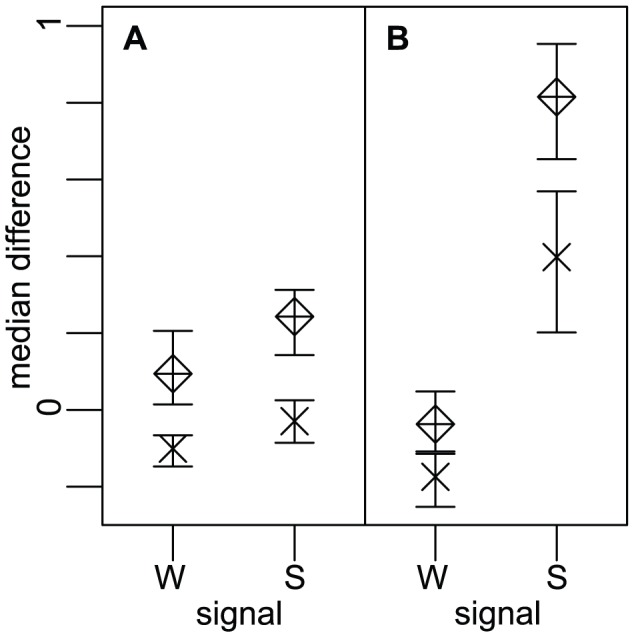
APA homozygotes have an increased gene expression for strong polyA signals and high miRNA score. Gene expression medians in several groups are shown: Median differences between the APA homozygotes and non-APA homozygotes (Rhombus), and between heterozygotes and non-APA homozygotes (Cross). 95% CI for median differences are shown. Expression of APA homozygotes is generally higher, followed by heterozygotes, and then finally non-APA homozygotes. (A): genes where alternative polyadenylation does not affect miRNA targeting (low miRNA score). Strong signals (S) have a slightly higher median difference compared to weak signals (W). (B): genes where alternative polyadenylation affects miRNA targeting (high miRNA score). Strong signals have a significantly higher median difference.

When comparing residual gene expression medians for the 3 genotypes (Fig. 7), we found that increased expression correlates with the number of APA alleles in the genotype and that SNPs with strong APA signal (S) had a significant gene expression median difference between the 3 genotypes (Fig. 7 A). This was particularly evident for SNPs with high miRNA score (Fig. 7 B), which are those that are supposed to have the strongest effect on miRNA regulation. Furthermore, a multiple regression on transcript length from the Burge RNA-seq data showed that APA homozygotes, cell proliferation, strong signals, and local and global GU levels, all contribute significantly to reduced transcript lengths (Table S5). Together, these results indicate that APA alleles of SNPs with strong APA sites and high miRNA scores can upregulate gene expression and link APA homozygotes to increased gene expression.

#### Gene expression can detect potentially functional APA-SNPs

Since genotype of SNPs in strong polyA sites and the loss of miRNA target sites can be associated with increased gene expression, we decided to use correlation to detect potentially functional APA-SNPs. Of the 333 candidate SNPs that mapped to gene probes, we discarded SNPs that were in genes whose maximum expression value among the 270 individuals was lower than the total expression median, to remove from the analysis genes that are very low or unexpressed in all the individuals. 243 SNPs remained and we tested these separately in a correlation analysis of genotype and mRNA expression.

We found 47 SNPs (on 47 genes) that were significantly different from 0 (see Table S6). All had a positive coefficient, indicating a positive correlation between genotype and gene expression. This fits both previous results where APA was associated with increased expression levels [4] and our RNA-seq results. After correcting for multiple testing with the Benjamini & Hochberg correction, 19 SNPs remained significant; 13 SNPs remained if correcting with stringent Bonferroni correction.

### Potentially functional APA alleles are positively correlated with risk alleles from disease-associated SNPs

Since SNPs can alter polyadenylation and affect miRNA target sites and gene expression, we wondered whether they can also play an important role in human diseases. We therefore tested if any of our APA-SNPs were linked to trait-associated SNPs from the NHGRI GWAS catalogue [20], [21], which consists of SNP-trait associations from published genome-wide association studies (GWAS) (accessed Apr. 18, 2011). Specifically, we mapped our 412 APA-SNPs to the 4304 GWAS SNPs, by using the mapping method described in Thomas *et al.* [22]. The mapping was based on linkage disequilibrium (LD) data from the Hapmap database (CEU population release 27). We identified 135 APA-SNP/GWAS-SNP pairs (consisting of 84 unique APA-SNPs and 123 unique GWAS SNPs) that had available haplotype data in Hapmap and one known and unique risk allele in the GWAS catalogue. For each APA-SNP/GWAS-SNP pair, we computed the correlation between the APA allele and risk allele as the LD value 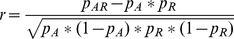 [23], where 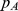, 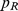, and 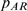 are respectively the APA allele frequency of the APA-SNP, the risk allele frequency of the GWAS SNP, and the “APA allele risk allele” haplotype frequency in the CEU Hapmap population. For each of the 84 unique APA-SNPs, we computed 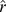 as the mean of 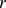 when an APA-SNP was linked to several GWAS SNPs, and similarly 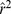 as the mean of 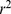.

We hypothesised that if APA-SNPs play a role in diseases, then APA alleles would be positively (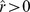) and strongly (high 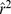) correlated with risk alleles, particularly for the significant APA-SNPs that we identified in the previous sections, as they are more likely to be functional, and particularly those that are linked to GWAS-SNPs from CEU-population-related studies, since the 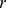 values are based on CEU haplotypes.

Among the 84 APA-SNPs, 60 were paired to GWAS-SNPs that are trait-associated in CEU-related populations. Nine of those SNPs were identified in the previous sections as significant APA-SNPs, and those nine SNPs had a significantly high number of positive 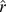 (more positive correlations between APA and risk alleles than expected) and a significantly high number of 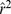 greater than 0.2 (higher number of correlations between APA and risk alleles than expected) (Table 2). In contrast, for 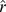 computed from CEU haplotypes but for GWAS-SNPs that are trait-associated in non-CEU-related populations, binomial test p-values were not significant, suggesting that GWAS and haplotype data should be matched according to population, to detect potential disease-related APA-SNPs.

**Table 2:** Potentially functional APA alleles are positively correlated with risk alleles from GWAS SNPs.

Predicate	Success count	Trial count	Success probability under 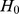	p-value ( 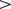 )
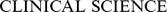	8	9	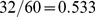	0.03
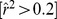	5	9	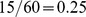	0.049

Two predicates were tested in a binomial setting: 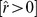 for positive trend correlation between APA and risk alleles, and 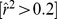 for the strength of the correlation. For the 60 APA-SNPs paired to GWAS-SNPs, the proportions of 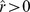 and 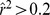 were respectively 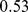 and 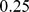. Among the 9 SNPs identified in the previous sections as functional candidate, respectively 8 and 5 succeeded the Bernoulli trial. Both null hypotheses were rejected.

Those results show that a significantly high proportion of our candidate SNPs is in LD with trait-associated SNPs and their APA alleles are positively correlated with risk alleles of trait SNPs. This suggests that those APA-SNPs can potentially be the cause of their corresponding disease-association signals measured and registered in the GWAS catalogue.

## Discussion

Our analyses confirmed the hypothesis (presented in Fig. 1) that SNPs can create functional alternative polyadenylation signals and thereby affect miRNA-based gene regulation and give increased gene expression. Both EST and RNA-seq analyses supported our hypothesis, despite some limitations. Additionally, the microarray analysis could further confirm these results and strengthen our hypothesis. Given the results from these three analyses, we estimate the proportion of functional APA-SNPs to be 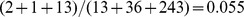 (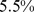).

The EST analysis supports our hypothesis but has some limitations. Specifically, we analysed EST data for 13 genes and found that 2 of them had an APA-SNP that could create polyA motifs and affect 3′UTR length. However, the EST analysis does not take into account the presence of a polyA tail in the EST sequence. Moreover, the ESTs came from a mix of tissues, which could also affect the results. Segregating ESTs based on tissue origin or filtering on sequences with clear tails in the “short” group, reduces sample size and affects statistical power. However, when combining sequences from our two significant genes, all of the 12 EST sequences ending at the alternative cleavage site and that have a polyA tail, had the APA allele. This number is significant (binomial test p-value of 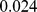, where the expected proportion of the APA allele is the combination of weighted allele frequencies of APA alleles for the 2 SNPs), and tells that the shortened transcripts arose from functional APA signals from the APA alleles.

Similarly, RNA-seq data from the Burge Lab, matched to miRNA expression data showed association between alternative polyA site strength (signal and GU-content), loss of miRNA target sites, allelic imbalance, and transcript expression. The Burge dataset was generated by using cDNA fragmentation, which gives a good coverage of 3′UTRs [24]. An increased allelic imbalance towards the APA allele could come from the loss of miRNA target sites, but also from the fragmentation method. This is because cDNA fragmentation gives a good coverage at the end of the transcript, and, in case of alternative polyadenylation, the transcript is shorter for the APA allele, which results in a high coverage at the SNP locus. In contrast, a longer transcript with the non-APA allele could have a higher coverage downstream, but a lower coverage at the SNP locus. Bias from cDNA fragmentation would therefore give an increased allelic ratio towards the APA allele simply because of transcript length differences. Consequently, we cannot exclude that some of the overall RNA-seq trends can be attributed to cDNA fragmentation bias.

The independent microarray data strongly support the EST and RNA-seq results, however. Specifically, the mRNA and miRNA microarray expression data showed association between alternative polyA site strength (signal and GU-content), loss of miRNA target sites, and transcript expression. This microarray analysis had the advantage of directly using genotype data from Hapmap, instead of genotyping SNPs through mapped RNA-seq reads. Furthermore, the microarray analysis focused on transcript expression differences between individuals and therefore required data from a unique cell type, whereas the RNA-seq analysis focused on allelic expression differences within a sample and could therefore involve different cell types. As expected, the microarray analysis showed similar results as the RNA-seq analysis, suggesting that the increased allelic ratios from RNA-seq data did not come from a potential bias due to the cDNA fragmentation method, but from the loss of functional miRNA target sites.

One clear disadvantage of using the RNA-seq data for genotyping and allelic-imbalance-based detection, was false positive homozygotes. We could detect potentially functional candidate SNPs by testing for allelic imbalance, which takes into account the number of reads and their quality, while testing for unusual allele proportion patterns. The difficulty was to find extreme allelic imbalance, as we could miss extreme imbalance by classifying a locus as homozygote because of too few reads (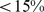) corresponding to the alternative allele. This was a conscious trade-off, however, since we wanted to maximise true positive heterozygotes and avoid false positives (*i.e.* predicted heterozygotes that were in fact homozygous).

RNA-seq data enabled us to genotype SNPs in expressed genes and compute allelic imbalance. Genotype classification could be checked with known genotypes from the Heap dataset and with mono-allelic SNPs. However the Heap dataset could not be used in the allelic imbalance analysis, because the library was generated by using RNA fragmentation, which gives a good coverage for the coding regions [24], but not for the UTRs. Since we were interested in SNPs in 3′UTRs, and particularly at the end of potentially alternative transcripts, RNA fragmentation would affect allelic imbalance.

The whole analysis is limited to SNPs that can make the reference 3′UTR shorter, lose miRNA sites and upregulate genes, because the loss of functional miRNA sites within the 3′UTR is more likely than the gain of new ones downstream of the annotated 3′UTR. However, it could be interesting to consider the hypothesis where SNPs in the signals at the end of the reference transcript could make 3′UTR longer having more miRNA target sites further downstream, and down-regulate the gene.

Alternative polyadenylation alleles play a role in 3′UTR shortening, gene deregulation, and increased disease risk (Fig. 1). Our analyses confirm that APA is an important factor for miRNA-mediated gene regulation [4]. EST data suggest that SNPs can create polyA motifs and affect 3′UTR length, and allelic imbalance from RNA-seq data coupled to miRNA expression data suggest an association between alternative polyA site strength (signal and GU-content), loss of miRNA target sites, allelic imbalance and transcript expression. Similarly, mRNA microarray expression data and matched genotypes of the same individuals, coupled with miRNA expression data could confirm association between alternative polyA site strength (signal and GU-content), loss of miRNA target sites, genotype and transcript expression.

Each of our analyses could also be used to detect potentially functional APA-SNPs. The detected APA-SNPs could further be linked to GWAS-SNP markers and a significant part of these APA-SNPs had their APA allele positively correlated with disease-risk alleles. We propose that these APA SNPs are potential disease-causative variants.

## Methods

### Datasets

We used SNP data from the CEU population (CEPH - Utah residents with ancestry from northern and western Europe) from the human haplotype map project (HapMap database [11]), release 22 for haplotype data, and release 27 for the genotype, allele, frequency, and linkage disequilibrium data. We used the human genome assembly version 18 (hg18) [25], RefSeq gene annotations (hg18 version), and EST sequences from the UCSC Genome browser [26]. We used human APA sites from PolyA_Db [12], [13]. We used disease-associated SNPs from the NHGRI GWAS catalogue [20], [21]. RNA-seq data came from Heap *et al.* [15] and from the Burge Lab [27]. Human miRNA profiles came from Landgraf *et al.* [16] (their Table S5) and from Wang *et al.* [18]. MicroRNA data came from the MirBase database release 16 [28].

### Candidate SNPs in alternative polyadenylation signals

Thirteen polyA signal motifs are known in human genes: AAUAAA, AUUAAA, UAUAAA, AGUAAA, AAGAAA, AAUAUA, AAUACA, CAUAAA, GAUAAA, AAUGAA, UUUAAA, ACUAAA, and AAUAGA [1] (ordered by strength ranks). We detected SNPs in potential APA signals, by a motif search that looks if any CEU Hapmap SNP in the 3′UTR of any coding gene would create/disrupt one of those 13 motifs. For a given SNP, the motif search looks for a given motif in an mRNA sub-sequence consisting of the SNP and its flanking sequences (6 nucleotides up/downstream), for each allele.

### PolyA_Db

We downloaded the 28.857 APA sites (human) from PolyA_Db [12], [13] from the UCSC track (hg18) [26]. We downloaded knownToLocusLink.txt and knownToRefSeq.txt from UCSC (hg18) [26] to convert entrez gene ID to RefSeq gene ID. We took the intersection between our APA signals and polyA sites from PolyA_Db, by taking all the sites from PolyA_Db that lie up to 40 bp downstream of our signals.

### EST

For each of the 13 candidate genes, we downloaded the EST sequences (Expressed sequence tag) from UCSC (hg18, tables ‘all_mrna’ and ‘all_est’) [26] that lie within their 3′UTR region. We also downloaded their alignment to their reference mRNA sequence from UCSC [26], and the list of EST that support the considered polyA site from PolyA_Db2 [13]. We used sequence alignment to identify the allele and haplotype of each sequence, when possible. Otherwise, the APA-SNP allele was imputed, by using haplotypes from the CEU Hapmap population [11] (see Dataset). We tested the proportion of APA alleles that support the candidate APA site, versus longer transcripts, by using a 2×2 contingency table. If the 4 expected values were greater than 5: we used the 2×2 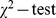, and Fisher's exact test otherwise.

### Allele imputation in EST data

Given a 3′UTR region of a gene of interest, we took all the phased SNPs from Hapmap [11] in that region, as well as their haplotypes in the CEU population [11]. For each of those SNPs, we identified the allele in the EST sequence when possible, to identify the EST haplotype. We discarded EST haplotypes that had zero identified allele. For each remaining EST haplotype, we selected haplotypes from Hapmap that fit the identified alleles in the EST haplotype. The APA-SNP could be imputed if there was only one unique allele at that SNP in all the selected haplotypes from Hapmap.

### RNA-seq data

We downloaded RNA-seq data from human primary 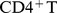 cells from 4 individuals [15] (Short read archive accession number: SRA008367), reads in FASTQ format, length of 45 bp. We downloaded Burge lab RNA-seq [27] (Short read archive: SRA002355, and Gene expression omnibus: GSE12946): Human tissue samples (brain, liver, heart, skeletal muscle, colon, adipose, testes, lymph node, breast, MAQC, 6 Cerebellum), immortalised and cancer cell lines (BT474, HME, MCF-7, MD435, T47D, MAQC UHR), reads in FASTQ format, length of 36 bp. MAQC is a mixture of brain cell types from several donors, MAQC UHR is a mixture of several cancer cell lines, and MD435 is thought to be contaminated by the M14 melanoma cell line. Therefore those 3 cell lines were discarded from the allelic imbalance analysis.

### RNA-seq mapping

We mapped RNA-seq reads using the RMAP software [29], with option ‘-Q’ for position weight matrix matching, based on quality score. Alignment was stored in BED files. We used the default options: 2 mismatches allowed in the 32 first nucleotides, 10 mismatches allowed in the whole read. Ambiguous reads were discarded. Paired-End reads were mapped as Single-End reads.

We mapped those reads to 3′UTR 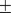
50 bp: the reference sequence is all 3′UTR DNA sequences (from the human genome assembly HG18 [25]) from all coding genes (excluding Y chromosome because of overlap with X), including introns, extended of 50 nucleotides up- and downstream. Overlapping sequences were merged (19012 regions). We mapped reads to a second version of the reference sequence, where reference alleles of APA-SNPs were replaced by non-reference alleles.

### RNA-seq genotyping

We counted base calls based on base quality probability score and sequence alignment score: We discarded reads mapped with an alignment score 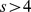, and reads that had a quality score 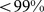 accuracy at the SNP. Quality score probability of accuracy at a SNP was computed as follows: 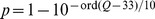, where 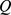 is the ASCII character of one base call in a read in FASTQ file format [30]. We computed the mapping score as 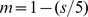, where 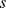 is the alignment score given by RMAP. We counted alleles as 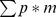 for each allele (for all the FASTQ files of each individual). We discarded alleles that do not fit Hapmap bi-allelic SNPs. If there was only one allele left, we classified the SNP as homozygous. If there were two alleles left, with both proportions greater than 0.15, we classified the SNP as heterozygous. If there were two alleles but one had its proportion lower than 0.15, we classified the SNP as homozygous with the allele having the biggest proportion.

### RNA-seq transcript end estimation

We mapped reads from the Burge dataset using the alignment software Bowtie [31] version 0.12.7 with default options. Bowtie generated alignments in the SAM format [32]. The transcript assembly software Cufflinks version 1.3.0 [33] was then used with the SAM files to generate a list of expressed exons for each run (default options). Those exons were then mapped back to annotated RefSeq genes. Exons that mapped to several different genes were discarded; the corresponding genes they overlapped were also discarded. For a given gene and a given run, the 3′ end of the exon that mapped the most downstream on the gene was used as an estimate of the gene's 3′ end. Finally, the distance between the estimate and the annotated transcript end was computed for each gene and each run. This distance 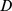 is negative when the transcript is shorter than the annotation and had a logarithmic distribution for negative 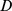
s. Few transcripts were longer than the annotated transcription end site, resulting in positive 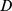 values. To handle these few positive 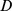 values, we put a threshold at 30, so that all 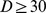 were truncated to 29. We then converted the 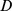
s to the logarithm scale by using the following formula: 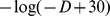.

### Allelic imbalance

Log Allelic Ratio for each heterozygous SNP is defined as 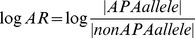, where counts of alleles are computed in a similar way as in the genotyping section (by taking base quality and alignment score into account). 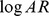 is positive when the transcripts with APA alleles are up-regulated compared to non-APA allele.

However, to avoid that a mapping bias towards reference alleles affects allelic ratios, we used a corrected allelic imbalance in our analyses, by combining allelic ratios computed from reads mapped to the reference genome with reference alleles, and allelic ratios computed from reads mapped to the same genome but with non-reference alleles at candidate SNPs. We defined it as the mean of the two log-ratios:


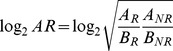


where 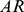 is the allelic ratio, 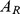 and 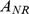 are the counts of APA alleles mapped to respectively the genome with reference alleles, and the one with non-reference alleles. Similarly 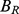 and 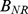 are the counts of non-APA alleles.

### GU-rich regions

We took all the known coding genes from the UCSC RefSeq gene database (hg18) [26]. To define the precise region of GU-analysis, for each gene, we computed the GU proportion in a 5-nucleotide long window sliding from the polyA signal downstream in a 70-nucleotide long region. Those curves represent the variation of GU proportion in the region for each gene. We then took the mean of all the curves, which showed that the increased GU region was from the 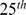 window to the 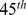 window (Fig. S4). We therefore defined the GU level as the mean of the GU-proportions in the 5-nucleotide windows, from the 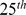 to the 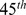 downstream of the polyA signal.

### Scoring APA for miRNA regulation

#### MicroRNA expression in Burge samples

Human miRNA profiles from Landgraf *et al.* [16] (their Table S5) were matched to Burge samples. We grouped and summed miRNA expression for mature miRNAs that have the same seed sequence and identified 117 seeds having a non-null expression.

#### MicroRNA expression in Hapmap cell lines

We took human miRNA profile from Wang *et al.* [18] (Gene expression omnibus: GSE14794), consisting of miRNA expression for EBV-transformed lymphoblastoid cell lines for 90 samples. For each of the 735 miRNA probes, we took the mean expression value among the 90 samples, resulting in one expression value per probe. We then computed the mean expression value among miRNA probes, and discarded all probes being smaller than the mean: 275 probes remained. We mapped probe IDs to miRNA seeds using the Illumina annotation file HumanMI_V1_R2_XS0000122-MAP. A total of 215 miRNA seeds remained. For each seed, we summed the exponential of expression values of the corresponding probes, since they were at a logarithm scale. We used these scores to compute the proportion of expression for each seed. We discarded seeds that do not have reference mature miRNAs in the MirBase database release 16 [28]. 163 seeds corresponding to 285 mature miRNAs remained.

#### MicroRNA scores

For each of the candidate SNPs and their corresponding RefSeq genes, we defined a short 3′UTR as the exonic region from the mRNA stop codon to the SNP, and a long 3′UTR, as the reference 3′UTR. We computed miRNA target predictions on those short and long sequences using the prediction tool from Saito *et al.* [34] for all mature miRNA sequence corresponding to the seed sequences identified in the considered cell line. The tool scores the mRNA/miRNA pairs, according to how a miRNA targets an mRNA: a high score means that the miRNA is more likely to down-regulate the mRNA. To compare scores for long and short UTRs, we normalised scores using the normalising method described in Thomas *et al.* [22]. Then for a given pair of miRNA seed and a UTR sequence, we took the score mean when one miRNA seed motif corresponded to several mature miRNAs, to have one score per seed. Then for a given UTR sequence, we computed a global score taking all expressed miRNAs into account: we summed scores for all the seeds, weighted by their proportion of expression in the considered cell line. When a gene corresponded to several RefSeq transcripts we took the score mean, resulting in having one long UTR score and one short UTR score for each candidate SNP. We could then compute the score difference for each SNP: this quantifies the potential effect of a cleavage site at the SNP locus on miRNA regulation.

### Messenger RNA expression and genotype

We downloaded human gene expression profiling of EBV-transformed lymphoblastoid cell lines from 270 unrelated Hapmap individuals [17] (Gene expression omnibus: GSE6536, data normalised across populations), and genotypes for the same individuals, from the Hapmap database release 27.

We mapped probe IDs to RefSeq genes using the BioConductor package for R [35], [36] (R version 2.10.1, AnnotationDbi package version 1.8.2 [37] and the annotation file illuminaHumanv1.db version 1.4.0). One candidate SNP could have one or several RefSeq gene IDs, which could be mapped to one or several probe IDs. Among those probe IDs, we selected the one with maximum variance across all the individuals in the dataset, and assigned it to the given SNP in the 3′UTR.

Genotype was encoded as 0, 1, and 2 for non-APA homozygotes, heterozygotes, and APA homozygotes, respectively.

### Bootstrapping median differences

We computed bootstraps of median differences: Given two groups with different sizes, we resampled with replacement in each group with their actual original size. We took the median in each resampling and computed the difference. We repeated this procedure 1000 times to create a median difference distribution, which was then used to compute the 95% confidence interval (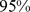 CI).

### Mapping APA-SNPs to GWAS

We mapped APA-SNPs to GWAS SNPs, using the mapping method described in Thomas *et al.* [22]. The mapping was based on linkage disequilibrium (LD) data from the Hapmap database (CEU population release 27). The mapping parameter was the threshold 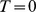 (see Thomas *et al.* [22]), to identify all neighbouring APA-SNP/GWAS-SNP pairs.

## Supporting Information

Figure S1 Diagram showing the workflow of our analyses and summarizing the number of SNPs investigated in each analysis.
(PDF)

Figure S2 Distribution of Hapmap SNPs within 3′UTRs of all RefSeq genes. Panel (A) shows the SNP distribution as a function of relative position within the 3′UTR (coding end site at position 0 and transcript end site at position 1). The SNP distribution, which is based on a kernel density estimate, is relatively uniform across the 3′UTR. Panels (B) and (C) show the SNP distribution from, respectively, 500 bp and 200 bp upstream of the transcription end position to the first 50 bp outside the gene. The SNP density is uniform within the 3′UTR except at the polyA signal position around 30 bp upstream of the transcript end.
(PDF)

Figure S3Distribution of distance 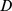 between estimated and annotated transcript ends within the Burge RNA-seq data, grouped into six sub-groups by the samples' cell proliferation state (non-proliferating vs. proliferating) and the APA SNPs' genotype (WT Hom.: homozygous wildtype; Het.: heterozygous; APA Hom.: homozygous APA). The distance 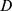 is shown on a negative logarithmic scale to reflect that the estimated transcript ends are shorter than the annotated ends. As expected, transcripts in proliferating cells are shorter than in non-proliferating cells. Moreover, transcripts that have homozygous APA SNPs are shorter than other genotypes; particularly for non-proliferating cells.(PDF)

Figure S4 GU content around transcription end site, based on all RefSeq genes. Mean of curves defined as GU proportion in a 5-nucleotide window sliding from the polyA signal to 70 nucleotides downstream. The GU-rich region is located between the 25th window and the 45th window.
(PDF)

Table S1 A portion of the EST-based polyA sites from PolyA_Db that do not have any signal in *N* nucleotides upstream of the cleavage site when looking at the reference genome, can be explained by a SNP in the region creating a signal from the SNP's non-reference allele.
(PDF)

Table S2Checking genotyping of 755 mono-allelic SNPs in 2 datasets (Heap and Burge). Columns correctHOM, incorrectHOM, and incorrectHET show the number and proportion of correctly classified homozygotes and of incorrectly classified homozygotes and heterozygotes among the total number of genotypes, respectively; ‘correct
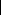
classified’ shows the proportion of correctly classified homozygotes among classified genotypes. Row Burge CEU corresponds to individuals in the Burge dataset that are Caucasian.(PDF)

Table S3 Genotyping results for the 412 candidate APA-SNPs in the Heap and Burge datasets.
(PDF)

Table S4 PolyA signal frequencies. The first three columns show polyA signal ranks, signal hexamers, and their frequencies in human genes from Tian *et al.* [1]; columns four and five show the hexamers' absolute and relative frequencies in human RefSeq 3′UTRs; column six shows the signal frequencies divided by the signals' relative frequencies in human 3′UTRs; and columns seven and eight show the counts and frequencies of our 412 candidate APA-SNPs. PolyA signal frequency (“PAS frequency”) corresponds well with how frequently the signal causes polyadenylation (“PAS frequency/Motif frequency”).
(PDF)

Table S5Multiple regression on distance between the estimated and the annotated transcript end (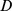; see Methods) and APA SNP genotype, cell proliferation status, APA signal strength, and local and global GU level. We only considered SNPs that lie at least 1500 kb from the annotated 3′ end. (A) All the dependent variables contribute significantly and negatively to the response variable (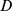), which means that homozygous APA SNPs, proliferating cells, strong signals, local and global GU levels all contribute to shortened 3′UTRs. (B) We get similar results when controlling for the global GU level. Specifically, the response variable in this analysis was the residuals from regressing global GU level on 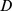.(PDF)

Table S6 Significant APA-SNPs from the microarray, EST and RNA-seq analyses.
(XLS)

Text S1 Translation of the Abstract into French by LFT.
(PDF)

Text S2 RNA-seq data successfully genotype known SNPs.
(PDF)
