# Supplementary material for: Single Nucleotide Polymorphisms Can Create Alternative Polyadenylation Signals and Affect Gene Expression through Loss of MicroRNA-Regulation
Source: PLoS Comput Biol. 2012 Aug 16;8(8):e1002621. doi: 10.1371/journal.pcbi.1002621 (PMC3420919; doi:10.1371/journal.pcbi.1002621)
Supplement: Figure S1 — Diagram showing the workflow of our analyses and summarizing the number of SNPs investigated in each analysis. (PDF) [file pcbi.1002621.s001.pdf]

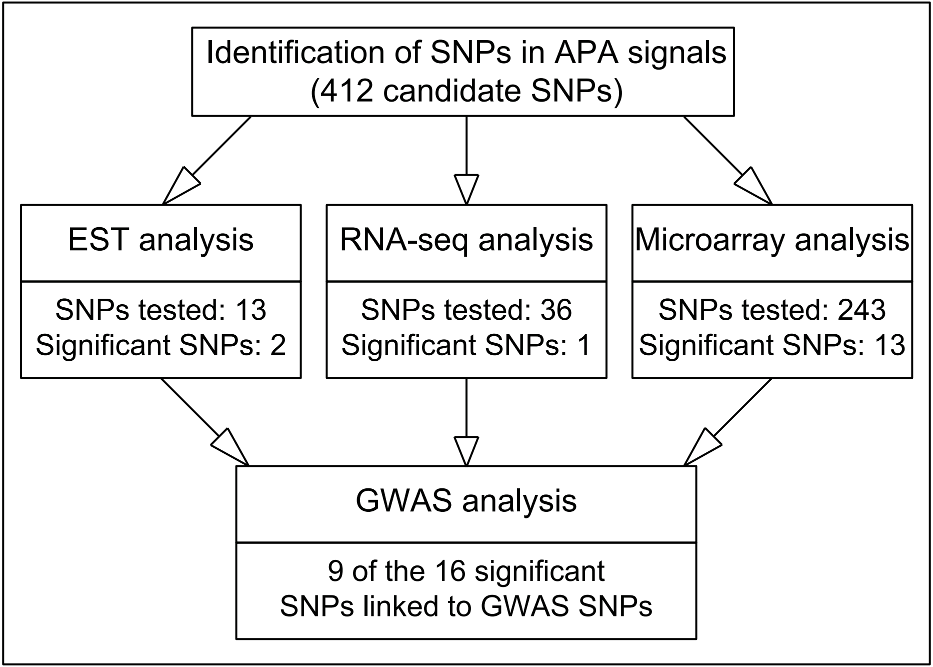
Identification of SNPs in APA signals (412 candidate SNPs)
EST analysis
SNPs tested: 13
Significant SNPs: 2
RNA-seq analysis
SNPs tested: 36
Significant SNPs: 1
Microarray analysis
SNPs tested: 243
Significant SNPs: 13
GWAS analysis
9 of the 16 significant SNPs linked to GWAS SNPs
