# Supplementary figures and images for: Single Nucleotide Polymorphisms Can Create Alternative Polyadenylation Signals and Affect Gene Expression through Loss of MicroRNA-Regulation
Source: PLoS Comput Biol. 2012 Aug 16;8(8):e1002621. doi: 10.1371/journal.pcbi.1002621 (PMC3420919; doi:10.1371/journal.pcbi.1002621)

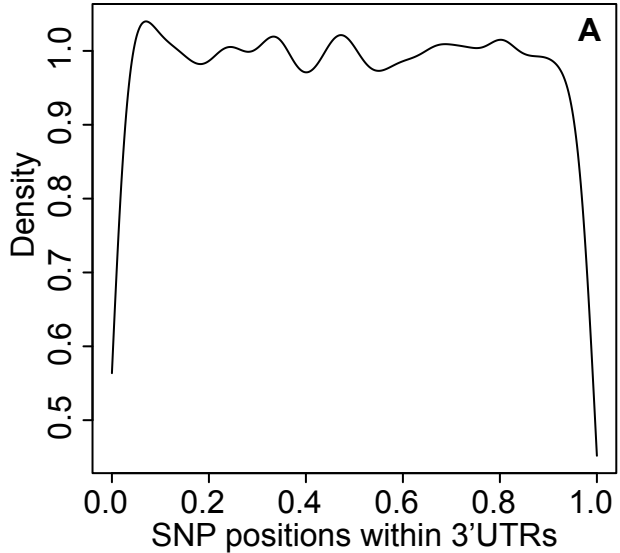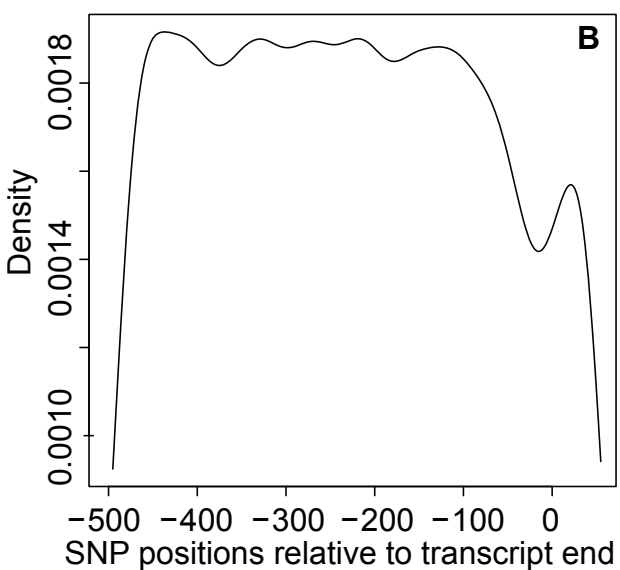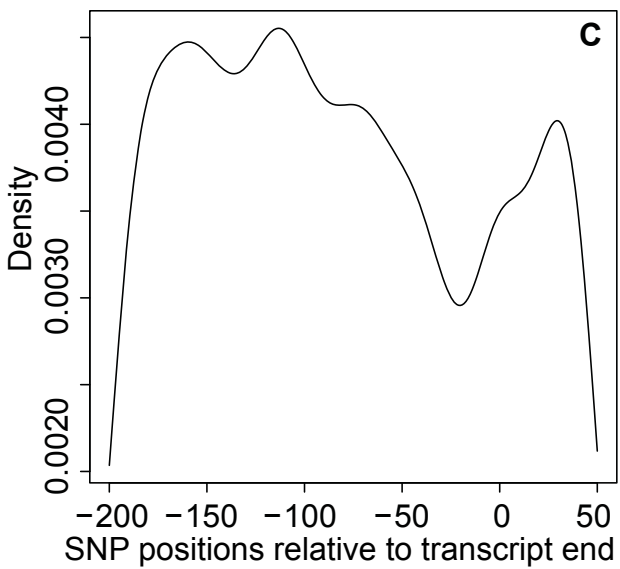

Supplement: Figure S2 — Distribution of Hapmap SNPs within 3′UTRs of all RefSeq genes. Panel (A) shows the SNP distribution as a function of relative position within the 3′UTR (coding end site at position 0 and transcript end site at position 1). The SNP distribution, which is based on a kernel density estimate, is relatively uniform across the 3′UTR. Panels (B) and (C) show the SNP distribution from, respectively, 500 bp and 200 bp upstream of the transcription end position to the first 50 bp outside the gene. The SNP density is uniform within the 3′UTR except at the polyA signal position around 30 bp upstream of the transcript end. (PDF) [file pcbi.1002621.s002.pdf]

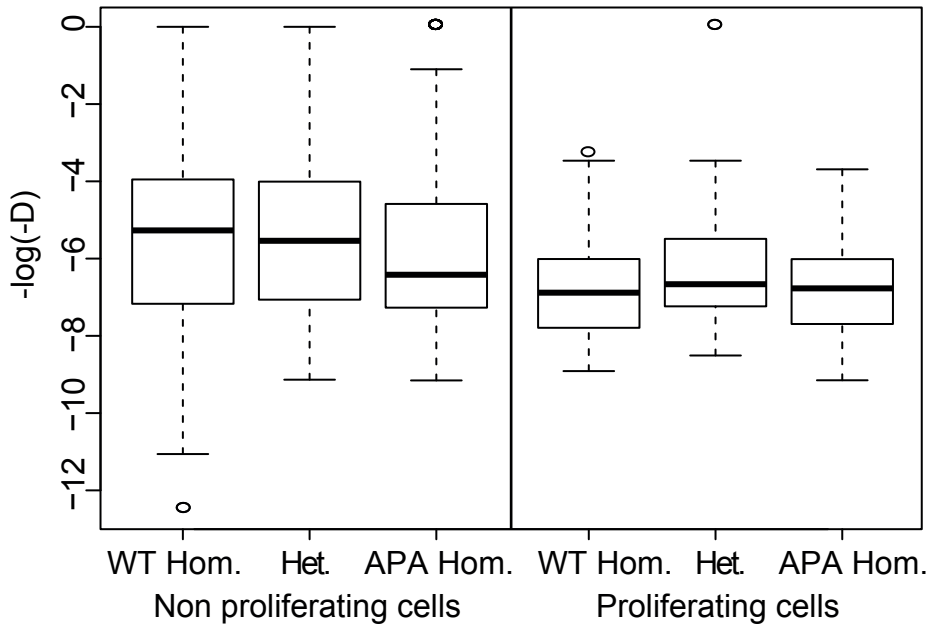

Supplement: Figure S3 — Distribution of distance between estimated and annotated transcript ends within the Burge RNA-seq data, grouped into six sub-groups by the samples' cell proliferation state (non-proliferating vs. proliferating) and the APA SNPs' genotype (WT Hom.: homozygous wildtype; Het.: heterozygous; APA Hom.: homozygous APA). The distance is shown on a negative logarithmic scale to reflect that the estimated transcript ends are shorter than the annotated ends. As expected, transcripts in proliferating cells are shorter than in non-proliferating cells. Moreover, transcripts that have homozygous APA SNPs are shorter than other genotypes; particularly for non-proliferating cells. (PDF) [file pcbi.1002621.s003.pdf]

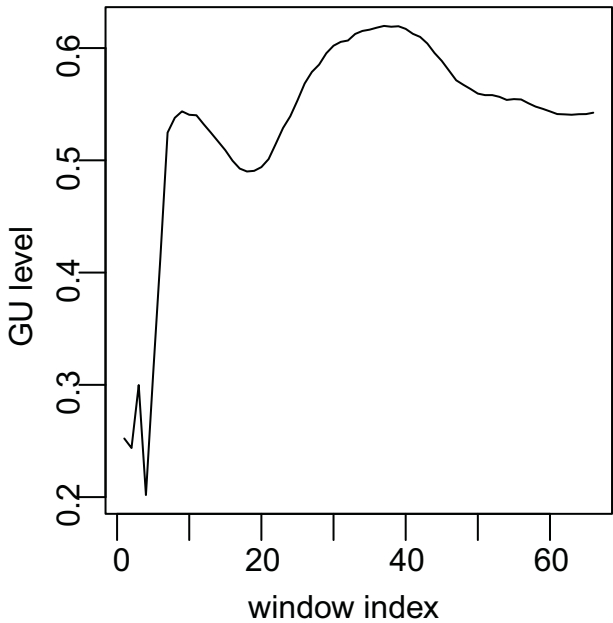

Supplement: Figure S4 — GU content around transcription end site, based on all RefSeq genes. Mean of curves defined as GU proportion in a 5-nucleotide window sliding from the polyA signal to 70 nucleotides downstream. The GU-rich region is located between the 25th window and the 45th window. (PDF) [file pcbi.1002621.s004.pdf]
