# Supplementary material for: Single Nucleotide Polymorphisms Can Create Alternative Polyadenylation Signals and Affect Gene Expression through Loss of MicroRNA-Regulation
Source: PLoS Comput Biol. 2012 Aug 16;8(8):e1002621. doi: 10.1371/journal.pcbi.1002621 (PMC3420919; doi:10.1371/journal.pcbi.1002621)
Supplement: Table S1 — A portion of the EST-based polyA sites from PolyA_Db that do not have any signal in N nucleotides upstream of the cleavage site when looking at the reference genome, can be explained by a SNP in the region creating a signal from the SNP's non-reference allele. (PDF) [file pcbi.1002621.s005.pdf]

Region Size	# PolyA sites	# PolyA sites with SNP-created Signal		
N	without Signal	CEU Hapmap	dbSNP126	dbSNP130
40	1728	6/1728	21/1728	24/1728
80	1343	9/1343	20/1343	26/1343
100	1210	10/1210	22/1210	26/1210
