# Supplementary material for: Single Nucleotide Polymorphisms Can Create Alternative Polyadenylation Signals and Affect Gene Expression through Loss of MicroRNA-Regulation
Source: PLoS Comput Biol. 2012 Aug 16;8(8):e1002621. doi: 10.1371/journal.pcbi.1002621 (PMC3420919; doi:10.1371/journal.pcbi.1002621)
Supplement: Table S2 — Checking genotyping of 755 mono-allelic SNPs in 2 datasets (Heap and Burge). Columns correctHOM, incorrectHOM, and incorrectHET show the number and proportion of correctly classified homozygotes and of incorrectly classified homozygotes and heterozygotes among the total number of genotypes, respectively; ‘correctclassified’ shows the proportion of correctly classified homozygotes among classified genotypes. Row Burge CEU corresponds to individuals in the Burge dataset that are Caucasian. (PDF) [file pcbi.1002621.s006.pdf]

Dataset	n	total genotypes	correctHOM	incorrectHOM	incorrectHET	correct|classified
Heap	4	$4 * 755 = 3020$	1650(54.6%)	7(0.23%)	3(0.1%)	99.4%
Burge	22	$22 * 755 = 16610$	5748(34.6%)	42(0.25%)	51(0.31%)	98.41%
Burge CEU	18	$18 * 755 = 13590$	4753(35%)	20(0.15%)	33(0.24%)	98.9%
