# Supplementary material for: Single Nucleotide Polymorphisms Can Create Alternative Polyadenylation Signals and Affect Gene Expression through Loss of MicroRNA-Regulation
Source: PLoS Comput Biol. 2012 Aug 16;8(8):e1002621. doi: 10.1371/journal.pcbi.1002621 (PMC3420919; doi:10.1371/journal.pcbi.1002621)
Supplement: Table S3 — Genotyping results for the 412 candidate APA-SNPs in the Heap and Burge datasets. (PDF) [file pcbi.1002621.s007.pdf]

Dataset	n	total genotypes	classified
Heap	4	$4 * 412 = 1648$	865(52.5%)
Burge	22	$22 * 412 = 9064$	3156(34.8%)
