# Supplementary material for: Single Nucleotide Polymorphisms Can Create Alternative Polyadenylation Signals and Affect Gene Expression through Loss of MicroRNA-Regulation
Source: PLoS Comput Biol. 2012 Aug 16;8(8):e1002621. doi: 10.1371/journal.pcbi.1002621 (PMC3420919; doi:10.1371/journal.pcbi.1002621)
Supplement: Table S4 — PolyA signal frequencies. The first three columns show polyA signal ranks, signal hexamers, and their frequencies in human genes from Tian et al. [1]; columns four and five show the hexamers' absolute and relative frequencies in human RefSeq 3′UTRs; column six shows the signal frequencies divided by the signals' relative frequencies in human 3′UTRs; and columns seven and eight show the counts and frequencies of our 412 candidate APA-SNPs. PolyA signal frequency (“PAS frequency”) corresponds well with how frequently the signal causes polyadenylation (“PAS frequency/Motif frequency”). (PDF) [file pcbi.1002621.s008.pdf]

rank	signal	PAS frequency	Motifs in 3'UTRs count	Motifs in 3'UTRs frequency	PAS frequency / Motif frequency	APA-SNPs count	APA-SNPs frequency
1	AAUAAA	53.18%	24436	15.90%	3.35	10	2.43%
2	AUUAAA	16.78%	13614	8.86%	1.89	27	6.55%
3	UAUAAA	4.37%	11434	7.44%	0.59	33	8.01%
4	AGUAAA	3.72%	7459	4.85%	0.77	23	5.58%
5	AAGAAA	2.99%	17767	11.56%	0.26	55	13.35%
6	AAUAUA	2.13%	9818	6.39%	0.33	23	5.58%
7	AAUACA	2.03%	7667	4.99%	0.41	42	10.19%
8	CAUAAA	1.92%	6507	4.23%	0.45	27	6.55%
9	GAUAAA	1.75%	5914	3.85%	0.45	23	5.58%
10	AAUGAA	1.56%	11005	7.16%	0.22	44	10.68%
11	UUUAAA	1.20%	25949	16.88%	0.07	55	13.35%
12	ACUAAA	0.93%	6570	4.27%	0.22	24	5.83%
13	AAUAGA	0.60%	5565	3.62%	0.17	26	6.31%
	total	93.16%	153705	100%		412	100%
