# Supplementary material for: Single Nucleotide Polymorphisms Can Create Alternative Polyadenylation Signals and Affect Gene Expression through Loss of MicroRNA-Regulation
Source: PLoS Comput Biol. 2012 Aug 16;8(8):e1002621. doi: 10.1371/journal.pcbi.1002621 (PMC3420919; doi:10.1371/journal.pcbi.1002621)
Supplement: Table S5 — Multiple regression on distance between the estimated and the annotated transcript end (; see Methods) and APA SNP genotype, cell proliferation status, APA signal strength, and local and global GU level. We only considered SNPs that lie at least 1500 kb from the annotated 3′ end. (A) All the dependent variables contribute significantly and negatively to the response variable (), which means that homozygous APA SNPs, proliferating cells, strong signals, local and global GU levels all contribute to shortened 3′UTRs. (B) We get similar results when controlling for the global GU level. Specifically, the response variable in this analysis was the residuals from regressing global GU level on . (PDF) [file pcbi.1002621.s009.pdf]

A

Variables	β_i estimates	p-values
Genotype (WT:0, HET:1, APA:2)	-0.30010	$5.9 * 10^{-12}$
Proliferating (True: 1, False: 0)	-0.89453	$< 2 * 10^{-16}$
Signal (Strong: 1, Weak: 0)	-0.18289	$1.7 * 10^{-2}$
Local GU level	-1.06154	$9.3 * 10^{-4}$
Global GU level	-10.30803	$1.5 * 10^{-4}$

Multiple R^2: 0.0726

B

Variables	β_i estimates	p-values
Genotype (WT:0, HET:1, APA:2)	-0.29752	$8.4 * 10^{-12}$
Proliferating (True: 1, False: 0)	-0.89865	$< 2 * 10^{-16}$
Signal (Strong: 1, Weak: 0)	-0.18015	$1.8 * 10^{-2}$
Local GU level	-0.93212	$1.9 * 10^{-3}$

Multiple R^2: 0.0616
