# Supplementary material for: Single Nucleotide Polymorphisms Can Create Alternative Polyadenylation Signals and Affect Gene Expression through Loss of MicroRNA-Regulation
Source: PLoS Comput Biol. 2012 Aug 16;8(8):e1002621. doi: 10.1371/journal.pcbi.1002621 (PMC3420919; doi:10.1371/journal.pcbi.1002621)
Supplement: Text S1 — Translation of the Abstract into French by LFT. (PDF) [file pcbi.1002621.s011.pdf]

Supporting Abstract:
Single Nucleotide Polymorphisms Can Create Alternative Polyadenylation Signals and Affect Gene Expression through Loss of MicroRNA-Regulation

Laurent F. Thomas and Pål Sætrom

Translation of the Abstract into French by LFT

La polyadénylation alternative (APA) est un mécanisme qui peut se produire par exemple lorsqu'un gène codant pour une protéine présente plusieurs signaux de polyadénylation (polyA) dans son dernier exon, résultant ainsi en ARN messagers (ARNm) de différentes longueurs au niveau de leur région 3 ' non traduite (UTR). Différentes longueurs de 3 ' UTR peuvent perturber la régulation des gènes par microARNs (miARNs) de telle sorte que l'expression des transcrits écourtés augmente. L'APA fait partie des mécanismes naturels de régulation des cellules humaines, mais semble également jouer un rôle important dans de nombreuses maladies humaines. Bien qu'une polyadenylation altérée dans le cadre de pathologies puisse avoir plusieurs causes, nous avons présupposé que des mutations d'ADN au niveau d'éléments particulièrement importants dans le processus de polyA, tels que le signal de polyA ainsi que la région en aval riche en GU, pouvaient être un important mécanisme d'altération. Pour tester cette hypothèse, nous avons identifié des polymorphismes nucléotidiques simples (SNP) qui peuvent créer ou perturber des signaux de polyA alternative (APA-SNP). En utilisant une approche d'intégration de données, nous montrons que les APA-SNPs peuvent affecter la longueur du 3 ' UTR, la régulation par miARN et l'expression d'ARNm — et ce, en comparant aussi bien l'expression des gènes d'individus homozygotes que l'expression allélique d'individus hétérozygotes. Par ailleurs, nous montrons qu'une proportion significative d'allèles causant l'APA est fortement et positivement liée aux allèles identifiées comme étant à risque par des études pangénomiques d'association à diverses maladies. Nos résultats confirment que l'APA-SNP peut modifier la régulation des gènes et que les allèles d'APA donnant des transcrits raccourcis ainsi qu'une augmentation de l'expression des gènes peuvent être une importante cause de maladies héréditaires.
