# Supplementary material for: Single Nucleotide Polymorphisms Can Create Alternative Polyadenylation Signals and Affect Gene Expression through Loss of MicroRNA-Regulation
Source: PLoS Comput Biol. 2012 Aug 16;8(8):e1002621. doi: 10.1371/journal.pcbi.1002621 (PMC3420919; doi:10.1371/journal.pcbi.1002621)
Supplement: Text S2 — RNA-seq data successfully genotype known SNPs. (PDF) [file pcbi.1002621.s012.pdf]

Supporting Result:
Single Nucleotide Polymorphisms Can Create Alternative Polyadenylation Signals and Affect Gene Expression through Loss of MicroRNA-Regulation

Laurent F. Thomas and Pål Sætrom

RNA-seq data successfully genotype known SNPs

We used our genotyping approach (see Methods) to analyse Heap and colleagues' RNA-seq data [1], which are based on human primary $CD4^+ T$ cells from 4 individuals. After mapping the reads to the reference genome, we could genotype our 755 candidate SNPs that are mono-allelic in the Hapmap CEU population, since the 4 individuals are known to be Caucasian. Of the $755 * 4 = 3020$ possible genotypes, 1650 were correctly classified as homozygous with the expected Hapmap allele, 1360 could not be classified because of the lack of reads (unexpressed genes), only 3 were misclassified as heterozygous, and 7 were misclassified as homozygous with the unexpected allele (minor allele frequency (MAF) allele) (Table S2). We also took the intersection between the known heterozygous SNPs reported in Heap *et al.* [1], and our candidate SNPs (26 genotypes, 19 SNPs), and could classify all of them as heterozygous (Table S2).

We also analysed the Burge Lab's RNA-seq data [2], which are based on 22 unrelated individuals; specifically, 7 cancer cell lines and 15 tissue samples. Again we genotyped SNPs that are mono-allelic in the CEU population and got similar results as for the Heap data (Table S2). Discarding samples that are not Caucasian increased the fraction of correctly classified genotypes (Table S2), which is consistent with us using the CEU Hapmap population to assess correctness. Specifically, by using the Hapmap CEU population to evaluate our genotyping approach, we got an upper-bound estimate of our method's accuracy, as the CEU population only approximates our samples' true genetic variations. Table S3 shows the number of classified genotypes in the 2 datasets for our candidate SNPs, which exclude mono-allelic SNPs. Based on the CEU-based validations, we expected most of these genotypes to be correct.

References

[1] Heap GA, Yang JHM, Downes K, Healy BC, Hunt KA, et al. (2010) Genome-wide analysis of allelic expression imbalance in human primary cells by high-throughput transcriptome resequencing. HUMAN MOLECULAR GENETICS 19: 122-134.

[2] Wang ET, Sandberg R, Luo S, Khrebtukova I, Zhang L, et al. (2008) Alternative isoform regulation in human tissue transcriptomes. NATURE 456: 470-476.
